# Determinants of COVID-19 vaccine Hesitancy: 2020 California Health Interview Survey

**DOI:** 10.1016/j.pmedr.2023.102200

**Published:** 2023-04-05

**Authors:** Ingyu Moon, Junghee Han, Keon Kim

**Affiliations:** a Alliance University (formerly Nyack College) School of Social Work, 2 Washington St. #2020, New York, NY 10004, USA; b University of Southern Indiana, Dept. of Social Work, 8600 University Boulevard, Evansville, IN 47712, USA

**Keywords:** Vaccine hesitancy, COVID-19, Determinants of health, Vaccine acceptance, Public health

## Abstract

Although the COVID-19 vaccine is a key intervention against the ongoing COVID-19 pandemic, vaccine hesitancy is a barrier to vaccination coverage, leading to a higher risk of COVID-19-related morbidity and mortality. To reduce vaccine hesitancy, the factors affecting it must be addressed. Based on the determinants of health approach, this study aimed to investigate whether the distribution of determinants of health differed between the vaccine hesitancy group and the vaccine acceptance group and to identify determinants of participants’ hesitancy to receive the COVID-19 vaccine if it was available. This study utilized the 2020 California Health Interview Survey Data collected between May and December 2020. Data were collected using a population-based web and telephone health survey. Data from 21,949 participants in California were included, and 4,183 (23.4 %) showed vaccine hesitancy. The following determinants were positively associated with vaccine hesitancy: female sex, Black and American Indian/Alaskan Native ethnicity, smoking, poverty, U.S.-born citizen, frequent use of social media, food insecurity, and limited healthcare access. Older age, not having severe psychological distress, not having diabetes, and high perceived safety in the neighborhood were negatively associated with COVID-19 vaccine hesitancy. This study also found that higher use of social media potentially threatened vaccine uptake, whereas frequent internet use was negatively associated with vaccine hesitancy. Public health and other relevant service providers should address the determinants of vaccine hesitancy and develop effective strategies and interventions for vaccine-hesitant groups.

## Highlights

• Social and individual factors are associated with COVID-19 Vaccine hesitancy.
• Higher use of social media potentially threatened vaccine uptake.
• Frequent general internet use was negatively associated with vaccine hesitancy.
• Considering determinants of health to promote vaccine uptake.

## 1 Introduction

Since coronavirus disease 2019 was first reported in December 2019 and declared a global pandemic by the World Health Organization on March 11, 2020 (Lone and Ahmad, 2020), the U.S. has experienced enormous challenges to healthcare systems and economies (Miller et al., 2020). Besides social distancing and face masks as strategies to prevent the spread of COVID-19 (Deng and Chen, 2022), vaccination is the best way to bring the pandemic under control and a beacon of hope for a return to normalcy (Sharun et al., 2020, Soares et al., 2021). The effectiveness and safety of the COVID-19 vaccine have been well-reported in a growing body of research, albeit to a greater or lesser extent based on risk group status and the type of vaccine (Lauring et al., 2022, Liu et al., 2021).

The U.S. Food and Drug Administration approved the first COVID-19 vaccine on August 23, 2020 (U.S. Food & Drug Administration, 2021). As of February 17, 2023, approximately 489 million COVID-19 vaccines had been given, and 220 million (67.2%) Americans received a second dose (Centers for Disease Control and Prevention [CDC], 2023a). The CDC has relaxed COVID-19 restrictions, such as wearing masks in public areas and schools and requiring a negative COVID-19 viral test result or documentation of recovery from COVID-19 for entering U.S. territories (CDC, 2022). COVID-19 vaccines have played a huge role in efforts to bring the pandemic under control (Altmann and Boyton, 2022).

Despite considerable initial enthusiasm and anticipation for COVID-19 vaccination, the vaccination program has been met with an undesirable phenomenon called “vaccine hesitancy” although it is not unusual (Yasmin et al., 2021). Vaccine hesitancy refers to the delay in acceptance of vaccination or refusal of vaccination despite its availability and safety, and it is influenced by contextual and individual factors (McKee and Bohannon, 2016). Vaccine hesitancy is a complex issue that can be influenced by a variety of factors, including individual, group, and vaccine-specific factors as well as broader contextual factors. These may include factors such as communication, media environment, historical influence, religion, culture, gender, socioeconomic status, politics, beliefs, attitudes about health, knowledge, costs, and personal, family, and community experience (MacDonald, 2015, Soares et al., 2021). COVID-19 vaccination intentions differ conceptually from traditional vaccine hesitancy, as the latter occurs when vaccines are widely available, and their safety is established, which is not yet the case with COVID-19 vaccination (Callaghan et al., 2021).

Vaccine hesitancy is identified as one of the top 10 global health threats (World Health Organization, 2019). In the country-specific simulation model, the mortality rate could be higher in countries with high COVID-19 vaccine hesitancy, which could be up to 7.6 times, than in countries with ideal COVID-19 vaccination uptake (Olivera Mesa et al., 2022). Vaccine hesitancy is a serious challenge to achieving high vaccination coverage against COVID-19, leading to a higher risk of COVID-19-related morbidity and mortality (Lazarus et al., 2022). According to a systemic review of 65 studies published in 2021, COVID-19 vaccine acceptance in the U.S. showed inconsistent results, ranging from 12% to 91.4% (Yasmin et al., 2021).

To examine vaccine hesitancy, we used Healthy People 2020′s determinants of health framework. Notably, the determinants of health are referred to as the non-medical factors that influence health outcomes, including biological, genetic, and psychological determinants (e.g., gender, race, sex, inherited conditions); social and environmental determinants (e.g., income, education, unemployment, food insecurity, exposure to media and internet, neighborhood safety, immigration status); health service determinants (e.g., insurance coverage, routine medical check-up); and individual behavior determinants (e.g., diet, physical activity, alcohol use, cigarette use; ODPHP, 2020). Besides the traditional determinants of health addressed in Healthy People 2020, the use of the internet and social media play key roles in public health and health promotion as determinants of health, particularly during the COVID-19 pandemic (Early and Hernandez, 2021, Zenone et al., 2023). Using determinants of health as a research approach to identify the sociocultural context in which an individual is born, matures, and ages may help diminish the prevalence of vaccine hesitancy (Gatwood et al., 2022).

Given the challenges of COVID-19 and barriers to vaccination, based on the determinants of health framework, alleviating vaccine hesitancy is the priority of the World Health Organization and the U.S. government. Understanding the factors associated with COVID-19 vaccine hesitancy is necessary to reduce vaccine hesitancy and consequently achieve high population immunity coverage (European Centre for Disease Prevention and Control, 2020). There must be a comprehensive understanding of factors associated with vaccine hesitancy and the groups who may be more likely to refuse vaccination. Because some previous studies on vaccine hesitancy related to COVID-19 have not employed a specific theoretical framework to present their findings, it has become challenging to categorize and address the factors involved. The study also focused on vaccine hesitancy in California in 2020 because California is one of the states that showed similar vaccine hesitancy rates (40.1%-50.0%) to the national average (44.6%; Campbell, 2021). As many factors contributing to vaccine hesitancy remain unchanged (Yasmin et al., 2021), investigating its determinants in 2020 would still be a valuable pursuit. Particularly, with the rise of public health misinformation through social media and anti-vaccine movements during that time (Burki, 2020), little is currently known about the determinants of COVID-19 vaccine hesitancy. Therefore, examining data from California in 2020 can provide valuable insights to inform interventions and policies in 2023 and beyond.

## 2 Methods

### 2.1 The California Health Interview Survey

This study utilized public data from the 2020 California Health Interview Survey (CHIS). The CHIS is an annual population-based web and telephone health survey for the noninstitutionalized population in all 58 counties of California (CHIS, 2021). It is a collaborative work of the UCLA Center for Health Policy Research, with multiple funding sources. Data were based on the self-report of adults 18 years and older. CHIS 2020 data collection reflected the COVID-19 situation, such as lockdown and stay-at-home orders throughout the 17 mailing waves (e.g., ensuring remote access to secure data). With rising COVID-19 cases, the questionnaire was updated in May 2020 to include a COVID-19 module, and data collected from May to December 2020 was used for this study (Ponce et al., 2021).

### 2.2 Measures

*Vaccine hesitancy (outcome variable).* The study used the following question to assess COVID-19 vaccine hesitancy: “If a vaccine becomes available for COVID-19, would you get it?” The answers were dichotomous: “yes (vaccine acceptance) = 0” or “no (vaccine hesitancy) = 1.”.

*Potential determinants of COVID-19 vaccine hesitancy (independent variables).* The variables considered potential determinants associated with vaccine hesitancy were selected and grouped based on the determinants of health framework from Healthy People 2020 (Table 1; ODPHP, 2020).

**Table 1:** Potential determinants on COVID-19 vaccine hesitancy.

**Determinants**	**Variables**	**Coding for analysis**
Biological/Genetic/psychological Factors	Age	3 levels of age groups (18–39 years, 40–69 years, 70–85 years)
	Gender	Self-reported gender (male vs. female)
	Race	6 racial/ethnic groups (White, Black, Latino, Asian, American Indian/Alaska Native, and other races)
	General Health Conditions	2 levels of health condition (good, very good, excellent VS. Fair and Poor)
	Asthma	Doctor ever told that you have asthma
	Heart Disease	Doctor ever told that you have any heart disease
	Diabetes	Doctor ever told that you have diabetes
	High Blood Pressure	Doctor ever told that you have high blood pressure
	Overweight (BMI =〈2 5)	WHO defined BMI (0 – 24.99 vs. 25 or higher)
	Psychological Distress	Having psychological distress in the past month (Kessler-6 score 0–12 vs. 13 or greater)
Social/ Environmental Determinants	Education	3 levels of educational attainment (under high school vs. high school diploma vs. college and above)
	Marital Status	Marital status with 3 categories (married vs. living with a partner/ widowed/ separated/ divorced vs. never married)
	Poverty	Living under the 100% Federal Poverty Line (0–99% FPL vs. 100% FPL and above)
	Food Security Status	Food security status 2 categories (Food security vs. food insecurity with/without hunger)
	Employment Status	Employment status with 2 categories (employed vs. unemployed)
	Neighborhood Safety	Frequency of feeling safe in the neighborhood with 4 levels (1–4; 1 = none, 2 = some, 3 = most, and 4 = all of the time)
	U.S. Citizenship	U.S. citizenship status 3 levels (U.S.-born citizen, naturalized citizen, and non-citizen)
Health Service Determinants	Medical Check-Up	Routine check-ups (within a year vs. never or more than a year)
	Health Insurance	Covered by any health insurance
Individual Behavior Determinants	Cigarette Smoking	Current smoker (not current smoker vs. current smoker)
	E-cigarette Smoking	Current e-smoker (not current e-smoker vs. current e-smoker)
	Eating vegetables	Number of times eating vegetables per week
	Illicit Drug Use	Use of heroin, methamphetamine, or prescription drug as not directed in the past year
	Frequency of Internet Usage	4-point Likert scale of frequency of internet usage, including streaming video/music, playing games, checking social media, using apps, browsing the web, etc. (1 = almost constantly, 2 = many times a day, 3 = a few times a day, and 4 = less than a few times a day)
	Frequency of Social Media Usage	4-point Likert scale of frequency of social media usage including Facebook, Instagram, Twitter, Youtube, etc. (1 = almost constantly, 2 = many times a day, 3 = a few times a day, and 4 = less than a few times a day)

### 2.3 Data analysis

Complex survey data analyses with the svy prefix command for Stata statistical software (version 13.0; StataCorp, 2013) were used to reduce possible bias and correct point estimates (StataCorp, 2021). For bivariate analysis, the study conducted Pearson chi-square and independent t-tests. Since the primary goal of this research was to predict categorical placement in COVID-19 vaccine hesitancy, a single binary dependent variable, based on 24 multiple independent variables, multiple logistic regression analysis was conducted to estimate the likelihood of hesitancy in vaccine uptake based on the possible determinants of health. Before the logistic regression, multicollinearity was tested. Next, the Archer–Lemeshow test, a modification of the Hosmer–Lemeshow test for complex survey data, was performed to check the goodness of fit of the model. The adequacy of the logistic model was also detected using the area under the receiver operating characteristic (ROC) curve, which contains information including the accuracy, sensitivity, and specificity of the logistic regression model. The ROC curve is equal to the concordance index, which can assess how good a model is at correctly classifying outcomes (Hosmer & Lemeshow, 2000). The concordance index can estimate the predictive power of the estimated model by comparing the estimated binary outcome with the observed outcome (Tesfaw and Fenta, 2021).

## 3 Results

This study reported a weighted percentage (Table 2) calculated based on design weight instead of an actual percentage calculated with the number of study participants due to the complex survey design nature. Out of 21,949 participants in California, 17,766 (76.6%) answered that they were willing to receive the COVID-19 vaccine if available, whereas 4,183 (23.4 %) participants would not. Table 2 shows the frequency distribution of total samples and groups of people with COVID-19 vaccine hesitancy and no hesitancy. Also, the mean and standard deviation of ordinal level variables were reported in Table 2.

**Table 2:** Frequency distribution and Bivariate Analysis of Potential Health Determinants and Vaccine Hesitancy among Californians.

**Item**	**Total** N (weighted %)	**Vaccine hesitancy** N (weighted %)	**No vaccine hesitancy** N (weighted %)	***p-value***
**BIOLOGICAL/GENETIC/PSYCHOLOGICAL DETERMINANTS**				
**Age** 18–39 40–69 70–85	4,849 (39.4) 11,931 (46.2) 5,169 (14.4)	1,081 (42.0) 2,391 (46.9) 711 (11.0)	3,768 (38.6) 9,540 (45.9) 4,458 (15.5)	0.000
**Gender** Male Female	9,575 (49.1) 12, 374 (50.9)	1,520 (43.9) 2, 663 (56.1)	8,055 (50.7) 9,711 (49.3)	0.000
**Race** White Black Latino Asian AI/AN^(1)^ Other races	13,697 (39.8) 778 (5.7) 2,328 (23.5) 2,755 (13.5) 147 (0.7) 2,244 (16.8)	2,211 (30.4) 310 (10.1) 658 (31.4) 362 (7.7) 52 (1.2) 590 (19.2)	11,486 (42.7) 468 (4.4) 1,670 (21.1) 2,393 (15.2) 95 (0.5) 1,654 (16.1)	0.000
**Health Conditions** Good/very good/excellent Fair/poor	19,303 (85.1) 2,646 (14.9)	3,650 (84.3) 533 (15.7)	15,653 (85.4) 2,113 (14.6)	0.185
**Asthma** No Yes	18,303 (83.9) 3,646 (16.1)	3,536 (84.7) 647 (15.3)	14,767 (83.6) 2,999 (16.4)	0.219
**Heart Disease** No Yes	19,822 (93.5) 2,127 (6.5)	3,871 (94.8) 312 (5.2)	15,951 (93.1) 1,815 (6.9)	0.004
**Diabetes** No Yes	19,582 (89.1) 2,367 (10.9)	3,755 (90.5) 428 (9.5)	15,827 (88.7) 1,939 (11.3)	0.024
**High Blood Pressure (HBP)** No Yes (including borderline HBP)	13,384 (67.2) 8,565 (32.8)	2,675 (68.9) 1,508 (31.1)	10,709 (66.6) 7,057 (33.4)	0.070
**Overweight** BMI =< 25 BMI > 25	5,212 (22.4) 16,737 (77.6)	917 (20) 3,266 (80)	4,295 (23.1) 13,471 (76.9)	0.010
**Psychological Distress** Kessler-6 score 0–12 Kessler-6 score 13 or greater	20,968 (93.8) 976 (6.2)	3,976 (94.2) 206 (5.8)	16,992 (93.7) 770 (6.3)	0.493
**SOCIAL/ENVIRONMENTAL DETERMINENTS**				
**Education attainment** Less than high school High school Collage and above	760 (15.2) 2,400 (21.8) 18,789 (63.0)	255 (22.6) 628 (25.8) 3,300 (51.6)	505 (12.9) 1,772 (20.6) 15,489 (66.5)	0.000
**Marital Status** Married Living with a partner Widowed/single/divorced/ Never married	11,935 (50.5)1,362 (9.5) 4,893 (14.6) 3,759 (25.4)	2,095(48.9) 289 (9.2) 1,032 (15.8) 767 (26.1)	9,840 (50.9) 1,073 (9.6) 3,861 (14.3) 2,929 (25.2)	0.371
**Poverty** Living under FPL 100% FPL and above	1,769 (13.2) 20,180 (86.8)	511 (17.3) 3,672 (82.7)	1,258 (12) 16,508 (88)	0.000
**Food Security Status** Food security Food insecurity	20,597 (89.6) 1,352 (10.4)	3,766 (85.5) 417 (14.5)	16,831 (90.9) 935 (9.1)	0.000
**Employment Status** Employed Unemployed, looking for work Unemployed, not looking for work	12,701 (64.4) 840 (6.1) 8,408 (29.4)	2,592 (65.5) 184 (6.8) 1,407 (27.7)	10,109 (64.1) 656 (5.9) 7,001 (30)	0.066
**U.S. Citizenship** U.S.-born citizen Naturalized citizen Non-citizen	17,356 (67.5) 3,402 (19.9) 1,191 (12.6)	3,290 (67.2) 631 (19.9) 262 (12.9)	14,066 (67.6) 2,771 (19.9) 929 (12.5)	0.915
**Neighborhood Safety** Mean (SD)	1.74 (0.68)	1.84 (0.67)	1.70 (0.68)	0.000
**HEALTH SERVICE DETERMINANTS**				
**Medical Check-Up** Within a year Never or more than 1 year	15,838 (67.9) 6,043 (32.1)	2,870 (65.1) 1,286 (34.9)	12,968 (69.8) 4,757 (30.2)	0.0002
**Health Insurance** Yes No	21,150 (92.8) 799 (7.2)	3,933 (90.1) 250 (9.9.)	17,217 (93.6) 549 (6.4)	0.000
**INDIVIDUAL BEHAVIOR DETERMINANTS**				
**Cigarette Smoking** Current smoker Not current smoker	1,190 (6.5) 20,759 (93.5)	297 (8.4) 3,886 (91.6)	893 (5.9) 16,873 (94.1)	0.0001
**E-cigarette Smoking** Current e-cigarette smoker Not current e-cigarette smoker	429 (2.8) 21,520 (97.2)	100 (3.7) 4,083 (96.3)	329 (2.4) 17,437 (97.6)	0.002
**Illicit Drug Use** Yes No	661 (3.5) 21,288 (96.5)	144 (3.7) 4,038 (96.3)	517 (3.4) 17,249 (96.6)	0.343
**Vegetable Consumption** Mean (SD)	9.68 (10.67)	9.55 (9.95)	9.73 (11.16)	0.555
**Frequency of Internet Usage** Mean (SD)	2.85 (0.95)	2.78 (0.86)	2.87 (0.94)	0.002
**Frequency of Social Media Usage** Mean (SD)	2.28 (1.02)	2.34 (0.82)	2.27 (1.05)	0.011

Note. (1) AI = American Indian, AN = Alaskan Native; p-values from corrected chi-square tests (age, gender, race, health conditions, asthma, heart disease, diabetes, HBP, overweight, psychological distress, educational attainment, marital status, poverty, food security, employment, citizenship, medical check-up, health insurance, cigarette, and e-cigarette smoking and illicit drug use); p-values from independent *t*-test (neighborhood safety, vegetable consumption, frequency of internet use and social media use).

### 3.1 Participants’ characteristics and differences in key variables between the vaccine hesitancy group and no hesitancy group

In our study, a larger proportion of young adults were hesitant to take it. Females were more hesitant than males (56.1% vs. 43.9%). Latinos and Blacks were more hesitant than other groups, with 31.4% of Latinos and 10.1% of Blacks expressing vaccine hesitancy, while 21.1% of Latinos and 4.4% of Blacks did not report hesitancy. People with heart disease or diabetes were less likely to be vaccine hesitant. Those with a BMI above 25 were more hesitant than those with a BMI of 25 or less (80% vs. 76.9%).

Statistically significant differences were found between vaccine-hesitant and non-hesitant individuals in terms of education, poverty, food security, and perceived neighborhood safety. More vaccine-hesitant individuals had some college education (66%) compared to the non-hesitant group (51.6%). A larger percentage of those below the poverty line were willing to get vaccinated (88%). Food insecurity was more prevalent among the vaccine-hesitant group, and they felt less safe in their neighborhood (t = 7.12, p =.000).

Within our sample, 67.9% of participants reported having received a medical check-up within the past year, and 92.8% reported having health insurance. Among those who were hesitant to receive the COVID-19 vaccine, 65.1% reported having a medical check-up within the past year, compared to 69.8% of those in the no-hesitancy group (22.1%). A higher proportion of respondents who were willing to receive the COVID-19 vaccine reported having health insurance (93.6%) compared to those with vaccine hesitancy (90%). Although few participants reported smoking cigarettes (6.5%) or e-cigarettes (2.8%), the vaccine-hesitant group had more smokers than the non-hesitant group. The vaccine-hesitant group also used the internet less frequently (t = -3.20, p =.002) but used social media more (t = -2.61, p =.011).

### 3.2 Effects of determinants of health on COVID-19 vaccine hesitancy

This study checked for multicollinearity before conducting logistic regression (VIF = 1.18). The model was statistically significant (F (34, 46) = 17.72, p =.000) and had a good fit according to the Archer-Lemeshow test (F (9,71) = 1.25, p =.2771) and the ROC curve analysis (0.66). Table 3 shows the logistic regression results of predictors on vaccine hesitancy. Age, gender, race, diabetes, and psychological distress were significantly associated with vaccine uptake. People aged 70–85 had 29.5% lower odds of hesitancy than those aged 18–39 (OR = 0.71, 95% CI = 0.571–0.87). Women had 34.5% greater odds of hesitancy than men (OR = 1.35, 95% CI = 1.20–1.50). Blacks, Latinos, AI/ANs, and other races had higher odds of hesitancy than Whites, while Asians had 31% lower odds (OR = 0.69, CI = 0.57–0.83). People with diabetes had 24% lower odds of hesitancy (OR = 0.76, CI = 0.62–0.93), and severe psychological distress was associated with lower odds of hesitancy (OR = 0.74, CI = 0.59–0.92).

**Table 3:** Estimated effects of selected determinants of health on COVID-19 vaccine hesitancy using multiple logistic regression model.

Vaccine	Odds Ratio (p-value)	Coefficient	Std. Err.	t	[95% Conf. Interval]	
**BIOLOGICAL/GENETIC/PSYCHOLOGICAL DETERMINANTS**						
**Age** (Ref. 18–39 years)						
40–69 years	0.91	0.09	0.07	−1.28	0.79	1.05
70–85 years	0.70***	0.35	0.07	−3.32	0.58	0.87
**Gender** (Ref. Male)						
Female	1.34***	−0.30	0.07	5.31	1.20	1.50
**Race** (Ref. White)						
Black	3.18***	−1.16	0.33	11.21	2.59	3.91
Latino	1.62***	−0.49	0.14	5.59	1.37	1.93
Asian	0.69***	0.37	0.07	−3.90	0.57	0.83
AI/AN^(1)^	2.85**	−1.04	0.92	3.24	1.50	5.44
Other races	1.43***	0.36	0.11	4.85	1.24	1.67
**Health conditions** (Ref., Good/very good/excellent)						
Fair/poor	0.91	0.09	0.07	−1.08	0.78	1.08
**Asthma** (Ref. No)						
Yes	0.91	0.09	0.06	−1.24	0.81	1.05
**Heart Disease** (Ref. No)						
Yes	0.89	0.11	0.10	−1.02	0.71	1.11
**Diabetes** (Ref. No)						
Yes	0.76**	0.27	0.08	−2.68	0.62	0.93
**High Blood Pressure** (Ref. No)						
Yes	1.00	−0.01	0.06	0.07	0.89	1.13
**Overweight** (Ref. BMI =<25)						
BMI > 25	1.01	−0.01	0.08	0.11	0.87	1.17
**Psychological Distress** (Ref. No or mild psychological distress)						
Severe psychological distress	0.74**	0.30	0.08	−2.78	0.59	0.92
**SOCIAL/ENVIRONMENTAL DETERMINANTS**						
**Educational Attainment** (Ref. Less than high school)						
High school	0.72**	0.33	0.08	−2.83	0.57	0.91
College and above	0.48***	0.72	0.04	−7.93	0.40	0.58
**Marital Status** (Ref. Married)						
Living with a partner	0.93	0.07	0.07	−0.88	0.80	1.09
Widowed/single/divorced						
Never married	0.86	0.15	0.07	−1.72	0.73	1.02
**Poverty** (Red. Living under FPL)						
100% FPL and above	0.89	0.11	0.08	−1.21	0.75	1.07
**Food Security Status** (Ref. Food security)						
Food insecurity	1.10	−0.94	0.11	0.97	0.91	1.33
**Employment Status** (Ref. Employed)						
Unemployed, looking for a job	0.91	0.09	0.11	−0.79	0.72	1.15
Unemployed, not looking for a job	0.95	0.06	0.06	−0.85	0.83	1.08
**U.S. Citizenship** (Ref. U.S.- born citizen)						
Naturalized citizen	0.92	0.09	0.07	−1.14	0.79	1.07
Non-citizen	0.67**	0.40	0.08	−3.32	0.53	0.85
**Neighborhood Safety**						
	0.84***	−1.81	0.03	−4.47	0.77	0.90
**Medical Check-Up** (Ref. Within a year)						
Never or more than 1 year	1.23***	0.21	0.07	3.66	1.10	1.39
**Health Insurance** (Ref. Yes)						
No	1.17	0.16	0.15	1.26	0.91	1.51
**INDIVIAUAL BHEAIOR DETERMINANTS**						
**Cigarette Smoking** (Ref. current smoker)						
Not current smoker	1.27*	0.24	0.08	−2.34	0.64	0.96
**E-cigarette Smoking** (Ref. current e-cigarette smoker)						
Not current e-cigarette smoker	1.50*	0.41	0.12	−2.18	0.46	0.97
**Vegetable Consumption**						
	1.00	0.01	0.01	0.38	0.99	1.01
**Illicit Drug Use** (Ref. Yes)						
No	0.97	−0.03	0.11	−0.3	0.77	1.21
**Frequency of Internet Usage**						
	0.87**	−0.14	0.03	−3.53	0.80	0.94
**Frequency of Social Media Usage**						
	1.09**	0.09	0.04	2.68	1.02	1.17

*Note. ^(1)^ AI =* American Indian, AN = Alaskan Native; *p < 0.05, **p < 0.01, and ***p < 0.001.

Education, citizenship status, neighborhood safety, and medical check-ups were significant factors in vaccine hesitancy. High school graduates had a 28.1% lower odds ratio (OR = 0.72, 95% CI = 0.57, 0.91) and those who attended college had a 51.6% lower odds ratio (OR = 0.48, 95% CI = 0.40, 0.58) compared to those without a high school diploma. Non-citizens had a 32.8% lower odds ratio than U.S. citizens (OR = 0.68, 95% CI = 0.52, 0.89), and people who felt safer in their neighborhood had an 0.83-fold lower odds ratio (OR = 0.83, 95% CI = 0.77, 0.90). Additionally, those who had not had a medical check-up within a year had a 23% lower odds ratio in vaccine hesitancy than those who had (OR = 1.12, 95% CI = 1.10, 1.39).

Cigarette smoking, e-cigarette use, internet usage, and social media usage were significant predictors of vaccine hesitancy among six individual behavior determinants, even after adjusting for covariates. Cigarette smokers had 27% higher odds of hesitating to get the COVID-19 vaccine, and e-cigarette smokers had 50% higher odds of hesitating than non-smokers (OR = 1.27, 95% CI = 0.64, 1.97 and OR = 1.50, 95% CI = 0.97, 2.32, respectively). A one-unit increase in internet use frequency was associated with a 13.1% decrease in vaccine hesitancy odds (OR = 0.87, 95% CI = 0.80, 0.94), whereas a one-unit increase in social media use frequency was associated with a 9% increase in vaccine hesitancy odds (OR = 1.09, 95% CI = 1.02, 1.17).

## 4 Discussion

Public concerns about aspects of the vaccine development process were widespread in 2020 (Tyson et al., 2020), even though the media constantly informed the public of scientists’ statements about the vaccine’s benefit and safety in combating infectious diseases and its usefulness for ending the pandemic (Ellyatt, 2020, Reynolds, 2020). This study found that throughout 2020, 23.4% of participants would not take a COVID-19 vaccine if available. This is similar to the Kaiser Family Foundation’s survey result that 27% of the public would not receive a vaccine even if it were free and scientists confirmed its safety (Hamel et al., 2020).

This study found higher odds for vaccine hesitancy among the following determinants of health: female gender, racial and ethnic minorities (Black, Latino, AI/AN, and other races), smokers (of both cigarettes and e-cigarettes), higher perception of neighborhood safety, and frequent use of social media. Lower odds for vaccine hesitancy were found for older individuals, Asians, individuals with diabetes or severe psychological distress, non-U.S. citizens, individuals who frequently used the internet, and highly educated individuals. Similarly, a systematic review of 65 studies found that the risk of being vaccine-hesitant was highly associated with younger age groups, females, and being Black (Yasmin et al., 2021). Young adults are more likely to choose not to receive a vaccine because they believe that COVID-19 is not a severe illness for young adults, as well as their concerns about side effects and mistrust of the vaccine’s efficiency (Adams et al., 2021). Similarly, women’s higher vaccine hesitancy is related to their belief that COVID-19 is not as risky to them (Liu and Li, 2021). Higher vaccine hesitancy among U.S. Black individuals is also more likely to be associated with mistrusting the U.S. government and institutions involved in vaccine production and promotion (Freimuth et al., 2017, Quinn et al., 2017). These explanations of higher vaccine hesitancy in certain groups indicate that targeting young adults, women, and Black people may increase the vaccine uptake rate more effectively than targeting their counterparts.

Individuals with a greater number of chronic health conditions had lower vaccine hesitancy (Warren et al., 2022). Consistent with this finding, our study also found that individuals with diabetes and heart disease had lower vaccine hesitancy. This suggests that individuals with certain chronic health conditions may be more informed about the benefits of vaccination and thus more willing to receive it. However, it is still unclear whether vaccine hesitancy varies across different types of chronic health conditions. For instance, diabetes was the only significant determinant associated with lower vaccine hesitancy, while other conditions such as heart disease, high blood pressure, and asthma were not (Mondal et al., 2021). Therefore, further research is needed to understand how vaccine hesitancy may differ across various chronic health conditions, in order to develop targeted interventions to increase vaccine uptake among these populations.

This study found that severe psychological distress was negatively associated with vaccine hesitancy. Unlike our finding, severe psychological distress was associated with higher vaccine hesitancy in a Japanese study (Okubo et al., 2021). Higher conspiracy beliefs about COVID-19 and vaccines among individuals with lower levels of psychological well-being may explain their findings (Roozenbeek et al., 2020). However, another study in England showed little indication of an association between coronavirus vaccine conspiracy beliefs and psychological distress (Freeman et al., 2022). Thus, further studies are needed on the association between psychological distress and vaccine hesitancy, along with the role of vaccine conspiracy and the susceptibility to misinformation of people with severe psychological distress.

Educational attainment was negatively associated with COVID-19 vaccine hesitancy. Compared to the group with less than high school attainment, groups with higher education reported lower vaccine hesitancy. Similarly, a lack of high school education was reported to be the most important determinant of COVID-19 vaccine hesitancy (Khairat et al., 2022). Individuals with higher levels of education are more likely to have greater information about the COVID-19 vaccine (Gerosa et al., 2021), which may lead to lower vaccine hesitancy (Khairat et al., 2022). Individuals who reported feeling safer in their neighborhoods were less likely to exhibit vaccine hesitancy. It may be related to the finding that disadvantage groups (i.e., racial minorities and lower income) were less likely to report feeling safety in their neighborhoods (not shown in tables). Interestingly, U.S.-born citizens are more likely to hesitate to get the COVID-19 vaccine than their counterparts. These findings suggest that knowledge and attitudes toward the vaccine may vary depending on social status and the community to which individuals belong.

Notably, increased use of social media was identified as a potential threat to vaccine uptake, whereas frequent internet use was negatively associated with vaccine hesitancy. Information sources contribute to knowledge gaps in the COVID-19 vaccine (de Vries et al., 2022, Gerosa et al., 2021). Individuals in the U.S. gather COVID-19-related information from an average of six sources, most frequently on social media and websites (Ali et al., 2020). Vaccine hesitancy has increased to some degree through misinformation about COVID-19 and vaccine side effects via traditional and social media (Gorman et al., 2022, Grimes, 2021, Van Der Linden et al., 2021). Some concerns have arisen regarding the information shared through social media due to harmful misinformation, which may be disseminated via the current anti-vaccination movement (Puri et al., 2020). While not all information shared on social media is false, the findings of this study suggest that social media may have contributed to the dissemination of misinformation to the public and amplified concerns about the COVID-19 vaccine. It's important to note that the spread of misinformation is a complex issue that involves multiple factors, including individual beliefs, social norms, and media literacy. Health authorities and stakeholders have openly addressed and discussed these false claims to prevent social media from spreading misinformation about the COVID-19 vaccine. Additionally, promoting health literacy education to enhance individuals' critical thinking skills and their ability to obtain accurate information is recommended.

Last, it is worth noting that the groups identified as “vaccine hesitancy” in this study may not necessarily be the same groups that, as of almost 3 years later, demonstrate lower vaccination rates. For example, our study, along with other previous studies, has found higher rates of vaccine hesitancy among females (Morales et al., 2022, Yasmin et al., 2021). However, the CDC reports that as of March 1, 2023, approximately 82.7% of females in the United States had received at least one dose of a COVID-19 vaccine, compared to 78.2% of males (CDC, 2023b). Also, our study's findings differ from the CDC's data, which indicate that Black and Hispanic individuals have reported higher vaccination rates (89.1% and 88.5%, respectively) than White individuals (87.1%; CDC, 2023b). This indicates that vaccine hesitancy is not a static issue, and various factors, such as successful vaccine approval and vaccine mandate policies, may impact individuals' vaccine hesitancy (Mello et al., 2022). As a result, changes in individuals’ COVID-19 vaccine uptake have occurred over time.

## 5 Limitations

The findings of this study should be interpreted with several limitations in mind. First, the study was conducted with cross-sectional data. This only allows examining correlations of factors, not causal inferences. Second, the study also relied on self-reported data that could be biased and not accurate. Third, vaccine hesitancy was assessed by only one item with a binary answer (yes or no): asking about willingness to receive the COVID-19 vaccine, which does not allow researchers to analyze more nuanced perceptions. Fourth, since the vaccine was released in December 2020, the responses reported in this study may not reflect recent feelings and beliefs regarding the COVID-19 vaccine, and actual receipt of the COVID-19 vaccine. Fifth, the proposed study only explored vaccine hesitancy of Californians. Therefore, it remains unclear whether the findings from California accurately represent other U.S. states. Sixth, the frequency of internet use is a compelling variable, as it has become an essential tool for work and life for many individuals. However, the way of measuring internet use in this study presents a challenge in interpreting what this variable truly represents and what aspects of internet use it encompasses. Last, this study does not take into account individual beliefs and perceptions, such as previous vaccination experience, perceptions of vaccine safety, and trust in healthcare. These psychological dispositions have been shown to play a significant role in vaccine hesitancy (Kricorian et al., 2022) and should be considered in future research on this topic.

## 6 Conclusion

Researching determinants of health is particularly important in the context of COVID-19 vaccine hesitancy. This study confirms that various determinants of health played an essential role in COVID-19 vaccine hesitancy. Specifically, individuals who identify as female, Black, or AI/AN, as well as those with lower educational attainment, income, and limited access to healthcare, exhibited higher levels of COVID-19 vaccine hesitancy. In addition, certain advantages in U.S. society, including being a U.S.-born citizen, younger age, and mentally and physically healthy, were also associated with vaccine hesitancy. Notably, the study also identified increased social media use as a potential threat to vaccine uptake, while frequent internet use was negatively associated with vaccine hesitancy. The insights gained from this study can inform the development of targeted interventions to address vaccine hesitancy and increase vaccine uptake, which is especially critical in the context of COVID-19 and future pandemics. From the findings of this study, policymakers and stakeholders may develop tailored messaging and outreach efforts to address concerns and misinformation among groups with higher levels of vaccine hesitancy, as well as efforts to improve access to vaccines and healthcare services in underserved communities. This study's findings can also help ensure that vaccines are accessible and acceptable to all, regardless of their socio-cultural background or circumstances. Future research should continue to address the role of social media in shaping vaccine hesitancy and explore additional determinants of health that contribute to vaccine hesitancy.

## 7 Ethics in Publishing

This research did not implement any experiments on humans and did not use data collected by the author. This research uses public data without identifiable private information. The Internal Review Board (IRB) at Alliance University approved this study. The data underlying this article were accessed from *https://healthpolicy.ucla.edu/chis/data/public-use-data-file/Pages/2019.aspx**. *UCLA Center for Health Policy Research publicly shared the derived data generated in this research.

## Funding

None.

## CRediT authorship contribution statement

**Ingyu Moon:** Conceptualization, Methodology, Software, Writing – original draft. **Junghee Han:** Data curation, Writing – review & editing, Visualization. **Keon Kim:** Writing – review & editing.

## Declaration of Competing Interest

The authors declare that they have no known competing financial interests or personal relationships that could have appeared to influence the work reported in this paper.

## Data Availability

Data will be made available on request.
